# Milk Drinking and Mortality: Findings From the Japan Collaborative Cohort Study

**DOI:** 10.2188/jea.JE20140081

**Published:** 2015-01-05

**Authors:** Chaochen Wang, Hiroshi Yatsuya, Koji Tamakoshi, Hiroyasu Iso, Akiko Tamakoshi

**Affiliations:** 1 Department of Public Health and Health Systems, Nagoya University Graduate School of Medicine, Nagoya, Japan; 1 名古屋大学大学院医学系研究科国際保健医療学・公衆衛生学; 2 Department of Public Health, Fujita Health University School of Medicine, Toyoake, Aichi, Japan; 2 藤田保健衛生大学医学部公衆衛生学; 3 Department of Nursing, Nagoya University School of Health Science, Nagoya, Japan; 3 名古屋大学大学院医学系研究科看護学専攻; 4 Public Health Department of Social and Environmental Medicine, Osaka University Graduate School of Medicine, Suita, Osaka, Japan; 4 大阪大学大学院医学系研究科社会環境医学講座公衆衛生学; 5 Department of Public Health, Hokkaido University Graduate School of Medicine, Sapporo, Japan; 5 北海道大学大学院医学系研究科社会医学講座公衆衛生学分野

**Keywords:** milk drinking, all-cause mortality, prospective study

## Abstract

**Background:**

Findings regarding the association between milk consumption and all-cause mortality reported by studies carried out in Western populations have been inconsistent. However, no studies have been conducted in Japan on this issue. The present study aimed to investigate the association of milk drinking with all-cause, cardiovascular, and cancer mortality in Japan.

**Methods:**

The data were obtained from the Japan Collaborative Cohort (JACC) study. A total of 94 980 Japanese adults aged 40–79 years who had no history of cancer, stroke, or chronic cardiovascular diseases were followed between 1988 and 2009. Multivariable-adjusted hazard ratios (HRs) and 95% confidence intervals (CIs) of mortalities were assessed using a Cox proportional hazard regression model and taking the lowest milk consumption group as the reference.

**Results:**

During a median of 19 years of follow-up, there were 21 775 deaths (28.8% and 35.3% from cardiovascular diseases and cancer, respectively). Drinking milk 1–2 times a month was associated with lower all-cause mortality in men compared to those who never drank milk (multivariable-adjusted HR 0.92; 95% CI, 0.85–0.99). In women, those who drank 3–4 times a week also had a lower mortality risk compared with those who never drank milk (HR 0.91; 95% CI 0.85–0.98). Inverse associations between drinking milk and mortality from cardiovascular diseases and cancer were found only in men.

**Conclusions:**

Drinking milk at least 1–2 times a month was associated with lower all-cause mortality in men compared to never drinking milk. An inverse association was also found between drinking milk and mortality from both cardiovascular diseases and cancer. However, lower all-cause mortality in women was found only in those who drank milk 3–4 times/week.

## Abstract (ja)

**【背景】:**

西洋人を対象に実施された牛乳摂取と総死亡率の関連性に関する研究の結果は一致しておらず、また、日本人においてこの関連は、まだ調べられていない。本研究の目的は、日本人の牛乳飲用と総死亡、循環器疾患死亡、がん死亡との関連性を調べることである。

**【方法】:**

JACC 研究の1988年から2009までの追跡データを用いた。がん、心血管疾患既往がなく、ベースライン時に40-79歳であった94,980人を解析対象者とした。牛乳を全く飲まない群を基準として、多変量調整ハザード比と95％信頼区間を、Cox比例ハザードモデルにより推定した。

**【結果】:**

中央値で19年の追跡期間中に、計21,775例の死亡が確認された（内、循環器疾患死亡が28.8%、がん死亡が35.3%）。牛乳を全く飲まない男性に比べて、月に1～2回牛乳を飲用する男性の総死亡率は有意に低かった（ハザード比：0.92、95%信頼区間：0.85 – 0.99）。また牛乳を全く飲まない女性に比べて、週に3~4回牛乳を飲用する女性の層死亡率は有意に低かった（ハザード比：0.91、95%信頼区間：0.85 – 0.98）。一方、牛乳飲用と循環器疾患死亡率やがん死亡率との負の関連性は、男性のみにおいて認められた。

**【結論】:**

牛乳を全く飲まない男性に比べて、月に1～2回以上飲用する男性では、総死亡率が有意に低かった。この関連性は、牛乳飲用と循環器疾患死亡率との負の関連性、牛乳飲用とがん死亡率との負の関連性と一致すると考えられた。一方、女性では、牛乳飲用と総死亡率との負の関連性は週3~4回摂取する群だけで認められた。

## INTRODUCTION

Milk is a widely consumed dairy product, rich in saturated fats, minerals, protein, and vitamins. The relationship of drinking milk or consumption of the nutrients found in milk and health has been often reported. For example, casein has been shown to have potent antimutagenic properties,^1^^,^^2^ and whey protein has been found to increase glutathione synthesis and suppress the development of tumors in an animal model.^3^ Calcium from dairy products suppressed colon tumorigenesis in one study^4^ and was associated with reduced mortality from stroke in another.^5^

However, on the contrary, some cohort studies have reported positive associations of milk consumption with stroke,^6^ coronary heart disease,^7^ endometrial cancer,^8^ ovarian cancer,^9^ and prostate cancer.^10^^,^^11^ Likewise, studies examining the association between milk consumption and all-cause mortality have produced inconsistent results. While an inverse association has been observed in a few studies,^12^^–^^14^ other investigations reported no association.^15^^–^^18^ In the baseline year of the present cohort study (1990), most “milk and dairy product” consumption (92.1%) was in the form of whole milk. However, the average milk consumption among Japanese at that time was much lower than that in Western countries.^19^

Therefore, we aimed to examine the association between milk consumption and mortality from all and major causes in a large-scale community-based cohort of Japanese men and women, incorporating a wide range of potential confounding variables.

## METHODS

The Japan Collaborative Cohort (JACC) Study for Evaluation of Cancer Risks, sponsored by the Ministry of Education, Sports, Science, and Technology of Japan, started between 1988 and 1990, enrolling subjects living in 45 areas throughout Japan. A total of 110 585 subjects (46 395 men and 64 190 women) 40–79 years of age completed self-administered questionnaires about their lifestyles and medical histories. Sampling methods and other details of the JACC study have been described elsewhere.^20^^–^^22^ Subjects with a previous history of any cancer, stroke, or chronic cardiovascular diseases (including myocardial infarction, angina pectoris, and other chronic ischemic heart disease) at baseline were excluded (*n* = 5693; 2493 men and 3200 women). Subjects who did not answer the question regarding milk consumption were also excluded (*n* = 9912; 4263 men and 5649 women). After these exclusions, 94 980 subjects (39 639 men and 55 341 women) were enrolled in the present analysis. The study design and informed consent procedure were approved by the Ethics Review Committee of Nagoya University School of Medicine.

### Follow-up and mortality surveillance

In each community, investigators conducted a systematic review of death certificates through 2009 for most communities (follow-up finished at the end of 1999 for 4 communities, 2003 for 4 communities, and 2008 for 2 communities). The date and cause of death were confirmed with the permission of the Director-General of the Prime Minister’s Office. Individuals who moved away from the study areas were treated as censored cases because subsequent deaths could not be confirmed. The registration of death is required by the Family Registration Law in Japan and is strictly observed. A few validation studies to date reported the accuracy of causes of death in death certificates.^23^^,^^24^ The Life Span Study showed that concordance between causes of death in death certificates and those of autopsy were 0.87 to 0.91 for cancer and 0.44 to 0.60 for cardiovascular disease.^23^ The Hisayama Study reported that concordance was 0.76 for all-cause mortality, 0.92 for cancer mortality, and 0.68 for cardiovascular mortality.^24^ We used the underlying causes of death coded by the *International Statistical Classification of Diseases and Related Health Problems-10th Revision* (ICD-10)^25^ to identify mortality endpoints: I00–I99 for mortality from cardiovascular disease (CVD) and C00–C97 for mortality from cancer.

### Statistical analysis

Information about milk drinking frequency and other lifestyle behaviors was obtained using a self-administered questionnaire. Subjects were divided into five groups by their self-reported milk-drinking frequency: “never,” “1–2 times/month,” “1–2 times/week,” “3–4 times/week,” and “almost every day” during the preceding year. The reproducibility and validity of the dietary questionnaire have been reported elsewhere.^26^ Specifically, the Spearman rank correlation coefficient between milk-drinking frequency and weighed dietary record for 12 days was 0.65, *P* < 0.001. We compared means using one-way analysis of variance and proportions using the chi-squared test.

Sex-specific age-adjusted all-cause, CVD, and cancer mortality rates were assessed according to the five categories of milk drinking frequency using the Poisson regression model and were expressed as the rate per 1000 person-years. For each subject, the person-years of follow-up were calculated from the date that the baseline questionnaire was completed until the time of death, moving out of the community, or the end of follow-up, whichever occurred first. Hazard ratios (HRs) and 95% confidence intervals (CIs) were calculated separately by sex using the Cox proportional hazard model adjusted for 5-year age groups.

In multivariate analyses, the models were adjusted for potential confounding variables, including smoking status (current, past, or never), alcohol drinking (current, past, or never), sleep duration (<7, 7–7.9, or ≥8 hours/day), body mass index (BMI, continuous), education level (attended school up to age 18), physical activity (exercise more than 1 hour per week), participation in health checkup in the preceding year, green-leafy vegetable intake (almost daily), history of hypertension, history of diabetes mellitus, and history of liver diseases. The number of categories of the covariates adjusted in the multivariate models was dichotomous (yes or no) if not specified. These variables were selected as covariates because they were known or suspected to confound the association.^5^^,^^27^^–^^30^ Missing values were treated as an additional category in the calculations.

Linear trends in mortality risks were assessed by assigning constants of 0, 1.5/30, 1.5/7, 3.5/7, and 1 to the ascending corresponding milk drinking frequency groups, and then treating the categories as numeric variables. Additionally, to address concerns regarding heterogeneity among study areas with different follow-up periods, meta-analysis using individual participant data was conducted. Specifically, three subgroups were defined as different cohorts: cohort 1 included subjects enrolled through 1999 (*n* = 12 944), cohort 2 included subjects enrolled through 2003 (*n* = 4345), and cohort 3 included subjects enrolled through 2008 or 2009 (*n* = 77 691). These cohorts were treated as strata in the stratified Cox regression model adjusted for the same covariates as the multivariate model. Heterogeneity between the cohorts was also tested.

Stratified analysis by sex and age (40–64 and 65–79 years) using the same models was performed because milk-drinking habits may significantly differ with advancing age. Interaction for age groups by milk drinking frequency was tested by adding the interaction term to the Cox models using a likelihood ratio test. In an attempt to avoid possible reverse causation, secondary analysis was also conducted using the same models and excluding deaths that occurred within 5 years of baseline (*n* = 4922; 2637 men and 2285 women). Statistical analyses were conducted with R version 2.15.3 (R Foundation for Statistical Computing, Vienna, Austria),^31^ using the Epicalc^32^ and survival packages.^33^ A *P* value of less than 0.05 was considered statistically significant.

## RESULTS

Baseline characteristics of the study sample are shown in Table 1. Subjects who drank milk almost every day (43.6% of the total sample; 40.3% of men, 45.9% of women) tended to be older among men; further, they were more likely to eat green-leafy vegetables every day, had a higher education level, and were less likely to be a current smoker in both men and women.

**Table 1.** Demographic characteristics at baseline according to milk intake frequency, 1988–1990, JACC Study

	Men (*n* = 39 639)							Women (*n* = 55 341)						
	
	Never	1–2 times/month	1–2 times/week	3–4 times/week	Almost everyday	*P*	Trend *P*	Never	1–2 times/month	1–2 times/week	3–4 times/week	Almost everyday	*P*	Trend *P*
Number of subjects	8551	3534	5962	5597	15 995			10 481	3666	7627	8146	25 421		
Age (years)	56.8 ± 9.9^a^	55.2 ± 10.1	55.5 ± 10.1	55.1 ± 9.9	58.2 ± 10.1	<0.01	<0.01	58.1 ± 10.2	56.6 ± 10.2	55.6 ± 10.1	55.6 ± 9.9	57.9 ± 9.9	<0.01	0.21
Height (cm)	162.6 ± 6.7	163.0 ± 6.7	163.2 ± 6.6	163.3 ± 6.6	163.1 ± 6.4	<0.01	0.03	150.5 ± 6.1	150.8 ± 6.3	151.3 ± 5.9	151.4 ± 5.6	151.4 ± 5.9	<0.01	0.05
Body mass index (kg/m^2^)	22.6 ± 3.4	22.8 ± 2.8	22.8 ± 2.8	22.9 ± 5.4	22.6 ± 2.7	0.01	0.51	23.0 ± 3.4	23.0 ± 3.8	23.1 ± 4.4	23.1 ± 3.1	22.8 ± 3.6	<0.01	0.25
Current smoker (%)	60.7	59.3	58.0	53.4	47.3	<0.01	<0.01	7.8	6.8	6.2	4.8	3.9	<0.01	<0.01
Current drinker (%)	75.3	78.7	77.8	79.1	74.0	<0.01	<0.01	22.0	24.3	25.9	27.5	24.7	<0.01	<0.01
Exercise^b^ (%)	19.0	26.6	25.1	25.7	29.6	<0.01	<0.01	13.6	17.0	18.5	18.8	22.6	<0.01	<0.01
Sleep duration^c^ (%)	30.0	33.5	31.9	32.4	32.0	<0.01	0.02	32.1	34.1	35.8	36.3	36.2	<0.01	<0.01
Green-leafy vegetables intake^d^ (%)	21.3	20.1	20.5	20.9	30.1	<0.01	<0.01	24.7	25.0	24.6	24.3	34.9	<0.01	<0.01
Physical checkup participation^e^ (%)	54.8	59.2	57.9	54.9	59.2	<0.01	<0.01	54.2	59.1	57.8	54.4	57.6	<0.01	<0.01
College or higher education (%)	9.8	12.4	12.7	11.1	15.9	<0.01	<0.01	4.8	6.5	7.2	6.6	9.4	<0.01	<0.01
History of hypertension (%)	18.5	17.5	17.1	16.9	18.7	0.03	0.45	21.6	20.5	19.1	19.0	20.1	<0.01	0.04
History of diabetes (%)	5.0	4.6	4.2	5.4	7.8	<0.01	<0.01	2.6	3.2	2.7	2.7	4.4	<0.01	<0.01
History of liver diseases (%)	5.8	6.4	6.1	5.4	7.2	<0.01	<0.01	3.5	5.0	3.9	4.0	5.0	<0.01	<0.01

^a^$\left(\bar{X}\right)$ ± SD (all such values).

^b^Defined as regular exercise >1 h per week.

^c^Percentage of subjects reported to sleep 7–8 h per day.

^d^Percentage of subjects reported to consume green-leafy vegetable almost everyday.

^e^Defined as participation in health checkups in the preceding year.

During the median 19 years of follow-up, 5538 subjects (2038 men and 3500 women) dropped out of the follow-up (5.8%), and 21 775 died (12 203 men and 9572 women). Of these deaths, 28.8% were from CVD (26.1% in men and 32.2% in women), and 35.3% were from cancer (38.5% in men and 31.2% in women). The five most common sites of cancer death were lung, stomach, liver, pancreas, and colon in men (28.9%, 22.8%, 13.5%, 7.4% and 6.5% of cancer deaths, respectively), and stomach, lung, pancreas, liver, and colon in women (19.7%, 13.7%, 12.1% 11.8%, and 11.6% of cancer deaths, respectively).

Age and multivariable-adjusted HRs of all-cause, CVD, and cancer mortality according to the five milk-drinking frequencies are shown in Table 2. Age-adjusted all-cause mortality rates seemed to become lower with increasing frequency of milk drinking in both men and women compared to subjects who never drank milk. In men, this inverse association of milk drinking frequency with all-cause mortality became insignificant after multivariable-adjustment (trend *P* = 0.09), although HRs for each category were still statistically significant. In women, both the trend and HRs for each category became insignificant after adjustment, except for the “3–4 times/week” category. Total CVD mortality rates were lower in men who drank milk 1–2 times/week or more compared to subjects who never drank milk: multivariable-adjusted HRs for each group compared to subjects who never drank milk were 0.86 (95% CI, 0.77–0.98) for the “1–2 times/week” group, 0.89 (95% CI, 0.79–1.01) for the “3–4 times/week” group, and 0.89 (95% CI, 0.82–0.98) for the “almost every day” group (*P* for trend = 0.06). Only women who drank milk “3–4 times/week” had a marginally significant lower risk of CVD mortality (HR 0.88; 95% CI, 0.78–1.01) compared to those who never drank milk.

**Table 2.** Hazard ratios for all-cause, cardiovascular, and cancer mortality by milk intake frequency, 1988–2009, JACC study

	Men (*n* = 39 639)						Women (*n* = 55 341)					
	
	Never	1–2 times/month	1–2 times/week	3–4 times/week	Almost everyday	Trend *P*	Never	1–2 times/month	1–2 times/week	3–4 times/week	Almost everyday	Trend *P*
**Person-years**	136 234	56 739	97 564	92 621	254 225		174 369	60 289	129 819	140 478	421 483	
**All-cause mortality**												
*Number of deaths*	2813	951	1669	1547	5223		2137	594	1215	1206	4420	
*Age-adjusted mortality rate*^a^	16.4	14.6	14.7	14.5	14.4		7.2	6.7	6.9	6.4	6.5	
*Age-adjusted HR (95% CI)*^b^	1	0.89 (0.82–0.95)	0.88 (0.83–0.94)	0.85 (0.79–0.90)	0.86 (0.83–0.91)	<0.01	1	0.97 (0.89–1.06)	0.95 (0.89–1.02)	0.88 (0.82–0.95)	0.91 (0.87–0.96)	<0.01
*Multivariable-adjusted HR (95% CI)*^c^	1	0.92 (0.86–0.99)	0.91 (0.85–0.96)	0.89 (0.84–0.96)	0.93 (0.89–0.98)	0.09	1	1.00 (0.91–1.05)	0.98 (0.91–1.05)	0.91 (0.85–0.98)	0.96 (0.91–1.01)	0.15
**Cardiovascular mortality**												
*Number of deaths*	733	272	423	406	1356		695	210	402	359	1419	
*Age-adjusted mortality rate*^a^	3.8	3.7	3.4	3.4	3.2		1.8	1.8	1.8	1.6	1.6	
*Age-adjusted HR (95% CI)*^b^	1	0.97 (0.85–1.12)	0.86 (0.77–0.97)	0.86 (0.76–0.97)	0.84 (0.77–0.92)	<0.01	1	1.09 (0.94–1.27)	1.01 (0.89–1.14)	0.85 (0.75–0.97)	0.92 (0.84–1.02)	0.01
*Multivariable-adjusted HR (95% CI)*^c^	1	0.98 (0.85–1.13)	0.86 (0.77–0.98)	0.89 (0.79–1.01)	0.89 (0.82–0.98)	0.06	1	1.14 (0.98–1.33)	1.03 (0.91–1.17)	0.88 (0.78–1.01)	0.99 (0.89–1.08)	0.32
**Cancer mortality**												
*Number of deaths*	1118	356	659	576	1994		624	159	373	407	1422	
*Age-adjusted mortality rate*^a^	7.3	6.0	6.4	5.9	6.3		2.9	2.4	2.7	2.7	2.8	
*Age-adjusted HR (95% CI)*^b^	1	0.84 (0.74–0.94)	0.88 (0.80–0.98)	0.79 (0.72–0.88)	0.86 (0.79–0.93)	<0.01	1	0.83 (0.70–0.99)	0.93 (0.82–1.06)	0.93 (0.82–1.06)	0.97 (0.88–1.06)	0.73
*Multivariable-adjusted HR (95% CI)*^c^	1	0.88 (0.78–0.99)	0.90 (0.82–0.99)	0.85 (0.76–0.94)	0.94 (0.87–1.01)	0.56	1	0.85 (0.72–1.02)	0.95 (0.83–1.08)	0.95 (0.84–1.08)	1.00 (0.91–1.11)	0.28

CI, confidence interval; HR, hazard ratio.

^a^Age-adjusted mortality was calculated using Poisson regression model and expressed as rate per 1000 person-years.

^b^Age-adjusted HR: adjusted for age categories (5-year age groups).

^c^Multivariable-adjusted HR: adjusted for age categories, smoking status, drinking status, physical activity, sleeping duration, body mass index, education level, participation in health checkups, green-leafy vegetable intake, and history of hypertension, diabetes, and liver disease.

Cancer mortality rates were lower in men who drank milk 1–2 times/month or more compared to those who never drank milk: multivariable-adjusted HRs for each category were 0.88 (95% CI, 0.78–0.99) for the “1–2 times/month” group, 0.90 (95% CI, 0.82–0.99) for the “1–2 times/week” group, 0.85 (95% CI, 0.76–0.94) for the “3–4 times/week” group, and 0.94 (95% CI, 0.87–1.01) for the “almost every day” group. However, we did not observe any linear trend between milk drinking frequency and cancer mortality in men (*P* for trend = 0.56). In women, milk drinking frequency was not associated with cancer mortality.

Table 3 shows the results of stratified analysis by subjects’ baseline age (40–64 and 65–79 years old). There were more subjects who drank milk every day in the older age group than in the younger age group (46.5% vs. 38.5% in men and 48.2% vs. 42.1% in women; data not shown in tables). Interaction for age groups by milk drinking frequency was marginally significant in men (*P* = 0.054) and significant in women (*P* = 0.022). All-cause mortality was inversely associated with the frequency of milk drinking in older men. Linearity of the association between milk drinking frequency and CVD mortality rates was not significant in either younger or older men, although the multivariable-adjusted HRs of younger men who drank milk “1–2 times/week” and “almost every day” compared to those who never drank milk were statistically significant: 0.81 (95% CI, 0.67–0.96) and 0.88 (95% CI, 0.77–1.00), respectively. Similarly, although the HRs for cancer mortality were <1 in older men who drank milk 1–2 times/month or more compared to those who never drank milk, the association was not linear.

**Table 3.** Multivariable-adjusted hazard ratios for all-cause, cardiovascular, and cancer mortality by milk intake frequency, stratified by age, 1988–2009, JACC study

	Men (*n* = 39 639)						Women (*n* = 55 341)					
	
	Never	1–2 times/month	1–2 times/week	3–4 times/week	Almost everyday	Trend *P*	Never	1–2 times/month	1–2 times/week	3–4 times/week	Almost everyday	Trend *P*
**Person-years**												
*Age 40–64 years at baseline*	112 528	48 023	81 867	78 349	200 197		133 281	48 339	107 514	116 059	325 453	
*Age 65–79 years at baseline*	23 706	8716	15 6978	14 273	54 028		41 089	11 951	22 305	24 419	96 030	
**All-cause mortality**												
*Age 40–64 years at baseline*												
Number of deaths	1473	518	892	851	2506		767	219	531	540	1714	
Age-adjusted mortality rate^a^	10.9	9.8	9.8	9.7	9.7		4.7	3.9	4.4	4.1	4.2	
Multivariable-adjusted HR (95% CI)^b^	1	0.98 (0.88–1.09)	0.93 (0.86–1.01)	0.93 (0.86–1.02)	0.97 (0.91–1.04)	0.76	1	0.89 (0.77–1.04)	0.97 (0.87–1.09)	0.89 (0.81–1.01)	0.95 (0.87–1.04)	0.55
*Age 65–79 years at baseline*												
Number of deaths	1340	433	777	696	2717		1370	375	684	666	2706	
Age-adjusted mortality rate^a^	57.1	49.9	51.2	50.1	49.1		29.7	29.6	29.1	26.9	27.0	
Multivariable-adjusted HR (95% CI)^b^	1	0.86 (0.77–0.95)	0.88 (0.80–0.96)	0.85 (0.78–0.93)	0.88 (0.83–0.94)	0.02	1	1.07 (0.96–1.21)	0.97 (0.89–1.07)	0.91 (0.83–1.00)	0.97 (0.90–1.03)	0.17
**Cardiovascular mortality**												
*Age 40–64 years at baseline*												
Number of deaths	351	122	183	201	551		195	61	137	132	433	
Age-adjusted mortality rate^a^	2.5	2.2	1.9	2.2	2.0		1.1	0.99	1.0	0.93	0.95	
Multivariable-adjusted HR (95% CI)^b^	1	0.97 (0.78–1.19)	0.81 (0.67–0.96)	0.93 (0.78–1.10)	0.88 (0.77–1.00)	0.19	1	0.99 (0.74–1.33)	1.00 (0.81–1.26)	0.89 (0.71–1.11)	0.96 (0.81–1.14)	0.59
*Age 65–79 years at baseline*												
Number of deaths	382	150	240	205	805		500	149	265	227	986	
Age-adjusted mortality rate^a^	16.2	17.2	15.8	14.7	14.5		10.4	11.4	10.9	9.0	9.6	
Multivariable-adjusted HR (95% CI)^b^	1	1.01 (0.83–1.22)	0.92 (0.78–1.08)	0.87 (0.73–1.03)	0.91 (0.81–1.03)	0.16	1	1.20 (0.99–1.45)	1.04 (0.90–1.21)	0.87 (0.74–1.02)	0.99 (0.89–1.11)	0.36
**Cancer mortality**												
*Age 40–64 years at baseline*												
Number of deaths	682	228	428	385	1132		328	84	233	244	760	
Age-adjusted mortality rate^a^	5.1	4.4	4.7	4.4	4.4		2.2	1.6	2.1	2.0	2.0	
Multivariable-adjusted HR (95% CI)^b^	1	0.94 (0.81–1.09)	0.96 (0.85–1.09)	0.91 (0.81–1.04)	0.96 (0.88–1.06)	0.67	1	0.80 (0.63–1.02)	0.98 (0.83–1.17)	0.93 (0.79–1.10)	0.99 (0.87–1.13)	0.55
*Age 65–79 years at baseline*												
Number of deaths	436	128	231	191	862		296	75	140	163	662	
Age-adjusted mortality rate^a^	18.7	14.9	15.2	13.8	15.9		6.9	6.3	6.3	6.8	6.9	
Multivariable-adjusted HR (95% CI)^b^	1	0.79 (0.65–0.97)	0.81 (0.68–0.95)	0.73 (0.62–0.87)	0.88 (0.79–0.99)	0.60	1	0.93 (0.72–1.19)	0.89 (0.73–1.09)	0.97 (0.80–1.17)	1.02 (0.89–1.17)	0.37

CI, confidence interval; HR, hazard ratio.

^a^Age-adjusted mortality was calculated using Poisson regression model and expressed as rate per 1000 person-years.

^b^Multivariable-adjusted HR: adjusted for age categories, smoking status, drinking status, physical activity, sleeping duration, body mass index, education level, participation in health checkups, green-leafy vegetable intake, and history of hypertension, diabetes, and liver disease.

Although we found lower age-adjusted all-cause and CVD mortality rates in women who drank milk 3–4 times/week compared to those who never drank in younger as well as older subgroups, HRs of milk drinking frequency with all-cause, CVD, and cancer mortality were not statistically significant after multivariate adjustment in any category in either age-group, including the “3–4 times/week” group. Results of the secondary analysis that excluded patients who died within 5 years of baseline were essentially the same (eTables 1 and 2).

## DISCUSSION

The present large prospective cohort study showed that men who drank milk at least 1–2 times/month had a significantly decreased risk of all-cause mortality compared to those who never drank milk. Although there was a suggestion of an inverse linear trend (*P* = 0.09), a dose-response relationship was not evident between milk drinking frequency of 1–2 times/month or more and all-cause mortality. The associations were similar between milk drinking and both CVD and cancer mortality in men. In women, the risk of all-cause mortality was lower in those with a milk drinking frequency of 3–4 times/week than those who never drank milk.

Consistent with the present finding, a previous meta-analysis reported a statistically significant inverse association between milk and dairy product consumption and all-cause mortality.^34^ However, another dose-response meta-analysis published recently reported a null association.^28^ Since these meta-analyses only included data from Western countries, the findings may not be readily applicable to East Asians, who tend to consume much less milk or dairy products. In Japan, even though most elementary schools and junior high schools started serving milk in the school meal in 1958 according to the School Lunch Program Act,^35^ the average daily consumption of milk per capita is still approximately one third of that in the United States.^36^ Therefore, even subjects who drank milk every day in the present study might only be counted as light-to-moderate milk drinkers in those Western studies, and differences in the absolute amount of milk intake may partly account for the inconsistencies. The present study would be the first to show that light-to-moderate milk consumption was associated with a lower risk of mortality from both CVD and cancer in men.

There are several possible explanations for the gender difference in the association of milk drinking with mortalities. First, women generally had lower-risk lifestyles than men regardless of milk drinking frequency, which might have obscured the potentially risk-lowering effect of milk drinking. Second, women would have received more advice to increase their calcium intake in an attempt to prevent osteoporosis,^37^ which might have modified their milk intake and calcium supplement use. This could have distorted the inverse association of milk drinking frequency with mortality in women since supplementation of calcium has been associated with significant lower blood pressure^38^ and lower mortality from ischemic heart disease.^39^ Third, the absolute numbers of deaths, as well as the age-adjusted mortality rates, were also higher in men than that in women, which may be related to greater statistical power to detect the association between milk drinking and mortality in men.

On the other hand, inverse associations of milk drinking frequency with all-cause mortality were more evident in the older age group in both sexes (*P* for interaction = 0.054 in men and 0.022 in women, Table 3 and eTable 2). A few explanations were speculated. First, cumulative (lifelong) milk intake would be higher in older subjects with milk drinking habit. Second, the potential risk-lowering effect of milk might be more visible in older individuals. For example, older people often have abnormal antioxidant status, such as lower erythrocyte glutathione reductase activity,^40^ and milk, being a rich source of riboflavin (vitamin B2), could function to protect tissues from oxidative injury.

Our findings may be interpreted in two ways. First, a milk-drinking habit may simply indicate a generally healthy lifestyle. For example, milk-drinking frequency was negatively associated with the prevalence of smoking habit and positively associated with higher education and the frequency of vegetable intake in the present study. Although these variables were adjusted for in the multivariable models, there is a possibility of residual confounding. Second, nutritional components from milk itself may possibly explain the present results. For example, milk minerals, especially calcium, potassium, magnesium, and phosphorus, were inversely associated with hypertension.^41^^–^^43^ Bioactive proteins and tripeptides from milk protein have also exerted beneficial effects in reducing blood pressure,^44^ cancer prevention,^2^ and enhancement of immune response.^45^ Angiotensin-converting enzyme (ACE) inhibition is one of the mechanisms that has been studied most in relation to the antihypertensive effect from milk-derived tripeptides.^46^ Results from *in vitro* experiments suggest that tripeptides derived from milk, such as isoleucine-proline-proline and valine-proline-proline,^47^ can inhibit ACE activity and potentially lower blood pressure. Furthermore, whey protein is rich in cysteine/cysteine and γ-glutamylcysteine dipeptides, which are efficient substrates for glutathione synthesis. Glutathione is a cellular antioxidant that destroys reactive oxygen species and detoxifies carcinogens.^48^ Milk fat also contains the highest level of naturally available conjugated linoleic acid among dietary sources, which could up-regulate the tumor suppressor gene in human breast cells^49^ and/or inhibit the expression of certain oncogenes.^50^^–^^52^ Other components in the milk, such as antioxidant vitamins (β-carotene, vitamin A, and vitamin D), may bind and/or solubilize potentially oxidizing fatty acids or other agents.

There are several limitations in the present study. First, information on milk drinking frequency and other lifestyles was self-reported and collected only at baseline. Second, types of milk (eg, reduced fat or whole milk), as well as the portion size per occasion, were not available from the FFQ used in the JACC study. However, milk drinking frequency was correlated with milk intake from 12-day weighed dietary records (*r* = 0.65, *P* < 0.001).^26^ Nevertheless, further studies with more accurate assessments of milk in terms of types and portion size are warranted. Third, end of follow-up differed by area (1999, 2003, 2008, and 2009) in the current study, which might have introduced some biases. However, additional meta-analysis using individual data did not indicate heterogeneity (*P* = 0.55 for men and 0.61 for women); HRs were similar to the original model (data not shown).

In conclusion, the present large-scale prospective study found a significantly decreased risk of all-cause mortality in Japanese men who drank milk at least 1–2 times/month compared to never drinking milk. In contrast, among women, drinking milk 3–4 times/week, not every day, was associated with lower all-cause mortality compared to never drinking milk.

## ONLINE ONLY MATERIALS

eTable 1. Hazard ratios for all-cause, cardiovascular, and cancer mortality by milk intake frequency, with exclusion of subjects who died during the 5 years of follow-up, 1988–2009, JACC study.

eTable 2. Multivariable-adjusted hazard ratios for all-cause, cardiovascular, and cancer mortality by milk intake frequency, stratified by age with exclusion of subjects who died during 5 years of follow-up, 1988–2009, JACC study.

Abstract in Japanese.
