# Supplementary material for: Milk Drinking and Mortality: Findings From the Japan Collaborative Cohort Study
Source: J Epidemiol. 2015 Jan 5;25(1):66–73. doi: 10.2188/jea.JE20140081 (PMC4275440; doi:10.2188/jea.JE20140081)
Supplement: eTable 1. [file je-25-066-s001.pdf]

eTable 1. Hazard ratios for all-cause, cardiovascular, and cancer mortality by milk intake frequency, with exclusion of subjects who died during the 5 years of follow-up, 1988-2009, JACC study

	Men (*n*=37,002)						Women (*n*=53,056)					
	Never	1-2 times/month	1-2 times/week	3-4 times/week	Almost everyday	Trend *p*	Never	1-2 times/month	1-2 times/week	3-4 times/week	Almost everyday	Trend *p*
**Person-years**	134,578	56,127	99,646	91,646	241,234		173,151	59,863	128,932	139,678	418,699	
**All-cause mortality**												
Number of deaths	2,362	784	1,435	1,292	4,385		1,869	509	1,061	1,068	3,857	
Age-adjusted mortality rate[a]	13.9	12.1	12.7	12.1	12.2		6.3	5.8	6.1	5.7	5.7	
Age-adjusted HR (95% CI)[b]	1	0.87 (0.79-0.94)	0.89 (0.84-0.96)	0.83 (0.78-0.89)	0.86 (0.82-0.91)	< 0.01	1	0.96 (0.87-1.06)	0.95 (0.88-1.02)	0.89 (0.82-0.96)	0.91 (0.86-0.96)	< 0.01
Multivariable-adjusted HR (95% CI)[c]	1	0.89 (0.83-0.97)	0.91 (0.86-0.98)	0.88 (0.82-0.94)	0.93 (0.89-0.98)	0.19	1	0.98 (0.89-1.09)	0.97 (0.89-1.04)	0.91 (0.83-0.98)	0.95 (0.90-1.01)	0.17
**Cardiovascular mortality**												
Number of deaths	612	227	351	343	1,140		606	180	349	315	1,248	
Age-adjusted mortality rate[a]	3.2	3.1	2.8	2.9	2.8		1.6	1.6	1.6	1.4	1.5	
Age-adjusted HR (95% CI)[b]	1	0.97 (0.83-1.13)	0.85 (0.74-0.97)	0.85 (0.75-0.97)	0.85 (0.77-0.93)	< 0.01	1	1.08 (0.92-1.28)	0.99 (0.87-1.14)	0.84 (0.74-0.97)	0.93 (0.84-1.02)	0.04
Multivariable-adjusted HR (95% CI)[c]	1	0.97 (0.83-1.13)	0.84 (0.74-0.96)	0.89 (0.78-1.01)	0.89 (0.81-0.99)	0.13	1	1.13 (0.96-1.34)	1.02 (0.89-1.16)	0.87 (0.76-1.01)	0.99 (0.89-1.09)	0.52
**Cancer mortality**												
Number of deaths	936	286	566	469	1,634		488	127	280	321	1 093	
Age-adjusted mortality rate[a]	6.2	4.9	5.6	4.9	5.3		2.5	2.0	2.3	2.4	2.4	
Age-adjusted HR (95% CI)[b]	1	0.80 (0.70-0.91)	0.89 (0.80-0.99)	0.76 (0.68-0.85)	0.85 (0.78-0.92)	< 0.01	1	0.80 (0.66-0.96)	0.91 (0.79-1.04)	0.92 (0.80-1.05)	0.94 (0.85-1.04)	0.94
Multivariable-adjusted HR (95% CI)[c]	1	0.84 (0.74-0.97)	0.91 (0.82-1.02)	0.81 (0.72-0.91)	0.93 (0.86-1.01)	0.55	1	0.82 (0.67-0.99)	0.92 (0.79-1.06)	0.93 (0.81-1.06)	0.97 (0.87-1.08)	0.61

CI, confidence interval; HR, hazard ratio.

[a] Age-adjusted mortality was calculated using Poisson regression model and expressed as rate per 1,000 person-years.

[b] Age-adjusted HR: adjusted for age categories (5-year age groups)

[c] Multivariable-adjusted HR: adjusted for age categories, smoking status, drinking status, physical activity, sleeping duration, body mass index, education level, participation in health checkups, green-leafy vegetable intake, and history of hypertension, diabetes, and liver disease
