# Supplementary material for: Milk Drinking and Mortality: Findings From the Japan Collaborative Cohort Study
Source: J Epidemiol. 2015 Jan 5;25(1):66–73. doi: 10.2188/jea.JE20140081 (PMC4275440; doi:10.2188/jea.JE20140081)
Supplement: eTable 2. [file je-25-066-s002.pdf]

eTable 2. Multivariable-adjusted hazard ratios for all-cause, cardiovascular, and cancer mortality by milk intake frequency, stratified by age with exclusion of subjects who died during 5 years of follow-up, 1988-2009, JACC study

	Men (*n*=37,002)						Women (*n*=53,056)					
	Never	1-2 times/month	1-2 times/week	3-4 times/week	Almost everyday	Trend *p*	Never	1-2 times/month	1-2 times/week	3-4 times/week	Almost everyday	Trend *p*
**Person-years**												
Age 40-64 years at baseline	111,786	47,642	81,358	77,756	198,855		132,708	48,131	106,964	115,585	324,116	
Age 65-79 years at baseline	22,792	8,486	15,289	13,889	52,379		35 856	10 271	19 346	21 625	84 364	
**All-cause mortality**												
Age 40-64 years at baseline												
Number of deaths	1,308	427	796	725	2,199		682	195	464	489	1,533	
Age-adjusted mortality rate[a]	9.8	8.2	8.8	8.3	8.7		4.2	3.5	3.9	3.8	3.8	
Multivariable-adjusted HR (95% CI)[b]	1	0.91 (0.82-1.02)	0.93 (0.85-1.02)	0.89 (0.81-0.97)	0.97 (0.90-1.04)	0.92	1	0.89 (0.74-1.05)	0.89 (0.78-1.01)	0.93 (0.82-1.05)	0.95 (0.86-1.05)	0.74
Age 65-79 years at baseline												
Number of deaths	1,054	357	639	567	2,186		1.187	314	597	579	2,324	
Age-adjusted mortality rate[a]	46.4	41.9	42.7	41.5	40.6		26.1	25.2	25.6	23.5	23.4	
Multivariable-adjusted HR (95% CI)[b]	1	0.88 (0.78-0.99)	0.89 (0.81-0.99)	0.85 (0.77-0.95)	0.89 (0.83-0.96)	0.06	1	1.05 (0.93-1.19)	0.97 (0.88-1.07)	0.90 (0.82-0.99)	0.95 (0.85-1.02)	0.12
**Cancer mortality**												
Age 40-64 years at baseline												
Number of deaths	605	187	384	326	988		290	72	193	215	668	
Age-adjusted mortality rate[a]	4.6	3.7	4.3	3.8	3.9		1.9	1.4	1.7	1.8	1.8	
Multivariable-adjusted HR (95% CI)[b]	1	0.87 (0.74-1.03)	0.97 (0.85-1.10)	0.87 (0.76-0.99)	0.96 (0.86-1.06)	0.78	1	0.77 (0.60-1.00)	0.92 (0.76-1.10)	0.93 (0.78-1.11)	0.98 (0.86-1.13)	0.42
Age 65-79 years at baseline												
Number of deaths	331	99	182	143	646		244	58	121	133	514	
Age-adjusted mortality rate[a]	14.7	11.8	12.1	10.5	12.3		5.8	4.9	5.5	5.6	5.4	
Multivariable-adjusted HR (95% CI)[b]	1	0.79 (0.63-0.99)	0.82 (0.68-0.98)	0.69 (0.57-0.85)	0.87 (0.76-0.99)	0.45	1	0.87 (0.65-1.16)	0.92 (0.74-1.15)	0.93 (0.76-1.16)	0.95 (0.81-1.11)	0.87
**Cardiovascular mortality**												
Age 40-64 years at baseline												
Number of deaths	308	102	159	172	488		174	59	125	122	393	
Age-adjusted mortality rate[a]	2.2	1.9	1.7	1.9	1.8		0.96	0.97	0.95	0.86	0.86	
Multivariable-adjusted HR (95% CI)[b]	1	0.92 (0.73-1.16)	0.79 (0.65-0.96)	0.89 (0.74-1.08)	0.89 (0.77-1.03)	0.37	1	1.08 (0.80-1.46)	1.03 (0.82-1.29)	0.91 (0.72-1.15)	0.97 (0.81-1.17)	0.58
Age 65-79 years at baseline												
Number of deaths	304	125	192	171	652		432	121	224	193	855	
Age-adjusted mortality rate[a]	13.3	14.6	12.8	12.5	12.0		9.2	9.5	9.4	7.7	8.4	
Multivariable-adjusted HR (95% CI)[b]	1	1.03 (0.84-1.28)	0.89 (0.73-1.07)	0.88 (0.73-1.07)	0.92 (0.79-1.05)	0.23	1	1.15 (0.94-1.42)	1.01 (0.86-1.19)	0.84 (0.71-1.01)	0.99 (0.88-1.12)	0.63

CI, confidence interval; HR, hazard ratio.

[a] Age-adjusted mortality was calculated using Poisson regression model and expressed as rate per 1,000 person-years.

[b] Multivariable-adjusted HR: adjusted for age categories, smoking status, drinking status, physical activity, sleeping duration, body mass index, education level, participation in health checkups, green-leafy vegetable intake, and history of hypertension, diabetes, and liver disease
