# Supplementary material for: Milk Drinking and Mortality: Findings From the Japan Collaborative Cohort Study
Source: J Epidemiol. 2015 Jan 5;25(1):66–73. doi: 10.2188/jea.JE20140081 (PMC4275440; doi:10.2188/jea.JE20140081)
Supplement: Abstract in Japanese. [file je-25-066-s003.pdf]

牛乳飲用と総死亡率：JACC 研究

王超辰[1]、八谷寛[1,2]、玉腰浩司[3]、磯博康[4]、玉腰暁子[5]

[1] 名古屋大学大学院医学系研究科国際保健医療学・公衆衛生学、[2] 藤田保健衛生大学医学部公衆衛生学、[3] 名古屋大学大学院医学系研究科看護学専攻、[4] 大阪大学大学院医学系研究科社会環境医学講座公衆衛生学、[5] 北海道大学大学院医学系研究科社会医学講座公衆衛生学分野

【背景】

西洋人を対象に実施された牛乳摂取と総死亡率の関連性に関する研究の結果は一致しておらず、また、日本人においてこの関連は、まだ調べられていない。本研究の目的は、日本人の牛乳飲用と総死亡、循環器疾患死亡、がん死亡との関連性を調べることである。

【方法】

JACC 研究の 1988 年から 2009 までの追跡データを用いた。がん、心血管疾患既往がなく、ベースライン時に 40-79 歳であった 94,980 人を解析対象者とした。牛乳を全く飲まない群を基準として、多変量調整ハザード比と 95%信頼区間を、Cox 比例ハザードモデルにより推定した。

【結果】

中央値で 19 年の追跡期間中に、計 21,775 例の死亡が確認された（内、循環器疾患死亡が 28.8%、がん死亡が 35.3%）。牛乳を全く飲まない男性に比べて、月に 1～2 回牛乳を飲用する男性の総死亡率は有意に低かった（ハザード比：0.92、95%信頼区間：0.85 – 0.99）。また牛乳を全く飲まない女性に比べて、週に 3~4 回牛乳を飲用する女性の層死亡率は有意に低かった（ハザード比：0.91、95%信頼区間：0.85 – 0.98）。一方、牛乳飲用と循環器疾患死亡率やがん死亡率との負の関連性は、男性のみにおいて認められた。

【結論】

牛乳を全く飲まない男性に比べて、月に 1～2 回以上飲用する男性では、総死亡率が有意に低かった。この関連性は、牛乳飲用と循環器疾患死亡率との負の関連性、牛乳飲用とがん死亡率との負の関連性と一致すると考えられた。一方、女性では、牛乳飲用と総死亡率との負の関連性は週 3~4 回摂取する群だけで認められた。
